# Linking Attainment of Wearable-Derived Sleep and Physical Activity Recommendations with Cardiometabolic Biomarkers and Employer Health Care Claims: A Feasibility Study

**DOI:** 10.2196/109259

**Published:** 2026-10-22

**Authors:** Gregory J Grosicki, William von Hippel, Finnbarr Fielding, Lucas Holden, Christian P Cheung, Jenna G Cohen, Kristen E Holmes

**Affiliations:** 1 Department of Performance Science and Health Outcomes, WHOOP, Inc., One Kenmore Square, Boston, MA, 02215, United States, 1 703-303-7812; 2 WHOOP Labs, WHOOP, Inc., Boston, MA, United States

**Keywords:** digital health, wearable technology, health economics, insurer costs, sleep, physical activity

## Abstract

Among 80 wearable members stratified into quartiles of attainment of combined physical-activity and sleep recommendations, higher attainment showed age-adjusted associations with lower resting heart rate and BMI and higher heart rate variability. The lowest-attainment quartile incurred approximately 12-fold higher annual per-member health care spending than the other quartiles.

## Introduction

Physical inactivity and poor sleep are associated with adverse health outcomes and higher health care costs [1,2]. Wearables are increasingly used to promote healthy behaviors [3,4]. Among WHOOP (WHOOP, Inc) members, more frequent wear has been associated with lower resting heart rate (RHR), higher heart rate variability (HRV), and healthier sleep and activity patterns [5]. Whether attainment of wearable-based sleep and physical activity recommendations is associated with markers of cardiometabolic health and health care use remains unclear [6].

We therefore examined, in a single-employer feasibility study, associations between attainment of WHOOP sleep and physical activity recommendations, cardiometabolic health, and employer-sponsored insurance claims.

## Methods

### Study Population

We studied 80 adults from a single US employer’s commercial health plan who were WHOOP members with 45 days or more of valid sleep and biometric data [7] during the 2024‐2025 plan year (June 1, 2024 to May 31, 2025; mean wear 294.9, SD 95.3 days). Age, sex, height, and weight were self-reported.

### Wearable Device and Measures

The WHOOP device uses photoplethysmography and accelerometry to quantify sleep, physical activity, and nocturnal RHR and HRV [8].

Physical activity is summarized as daily Strain, a logarithmic score (0‐21) quantifying cardiovascular load from the continuous heart rate signal using personalized intensity zones and time spent in each. Members receive a personalized daily Strain recommendation derived from the morning Recovery score, which combines nightly RHR, HRV, and respiratory rate, each relative to the member’s baseline, with Sleep Performance; recommendations are higher when Recovery is higher. Sleep Performance is a 0 to 100 score reflecting sleep obtained relative to an individual’s personalized sleep needs. A multicomponent revision (adding consistency, efficiency, and stress) was introduced in mid-April 2025.

WHOOP heart rate and 2-stage sleep classification demonstrate 99% agreement with electrocardiography and 86% to 89% agreement with polysomnography [9,10].

### Physical Activity and Sleep Recommendation Attainment

For each participant, we calculated a composite recommendation-attainment score as the mean of 2 age-adjusted, standardized (*z*-scored) measures: Strain-recommendation attainment (percentage of wear days meeting the personalized Strain recommendation) and Sleep Performance. Components were weighted equally to represent joint attainment of both recommendations and were fixed before any claims data were received, as the insurer required predefined groups. Participants were ranked into quartiles of the composite score (Q1=lowest to Q4=highest; n=20 in each quartile).

### Outcomes

Primary cardiometabolic outcomes were nocturnal RHR, HRV (root mean square of successive differences), and BMI. While these outcomes are not used in the composite score directly, RHR and HRV inform the Strain recommendation as within-person deviations from baseline; in contrast, self-reported BMI is algorithm independent. Claims were provided only as monthly medical and pharmacy expenditures aggregated by quartile and indexed by payment month, which extended beyond the service window because of runout. Per-member-per-year (PMPY) cost was calculated as total paid claims divided by 20.

### Statistical Analysis

Age-adjusted monotonic trends across recommendation-attainment quartiles were evaluated using 2-sided Jonckheere-Terpstra tests. We calculated linear regression estimated age-adjusted effect sizes per SD increase in recommendation attainment, as well as partial Spearman correlations. Sensitivity analyses recalculated the composite from prerevision data (quartile agreement by weighted κ) and examined each component separately (Multimedia Appendix 1). Because claims were quartile-level totals, costs are presented descriptively, with sensitivity analyses excluding catastrophic claim months (>US $100,000). Analyses were performed in R (version 4.4; R Foundation for Statistical Computing) with *α*=.05.

### Ethical Considerations

All participants provided study-specific electronic informed consent for research use of deidentified data, and the protocol was approved by the Salus Institutional Review Board (protocol number 25724).

## Results

Participants (N=80; 20 per quartile) had a mean age of 34.2 SD (SD 8.7) years and a mean BMI of 25.6 (SD 3.9) kg/m²; 18 participants (22.5%) were female. Mean age varied across quartiles (29.8‐36.1 years; Table S1 in Multimedia Appendix 1).

RHR decreased with attainment (Figure 1; J-T, *P*=.001; −3.6 beats per minute [bpm] per SD, 95% CI −5.4 to −1.8), as did BMI (J-T, *P*=.005; −1.0 kg/m² per SD, 95% CI −1.8 to −0.2). HRV increased with attainment (J-T, *P*=.011; +9.1% per SD on the log scale, 95% CI −0.5% to 19.5%). Partial Spearman correlations were significant for RHR (ρ = −0.34, *P*=.002), HRV (ρ=.25, *P*=.02), and BMI (ρ = −0.36, *P*=.001).

**Figure 1.**
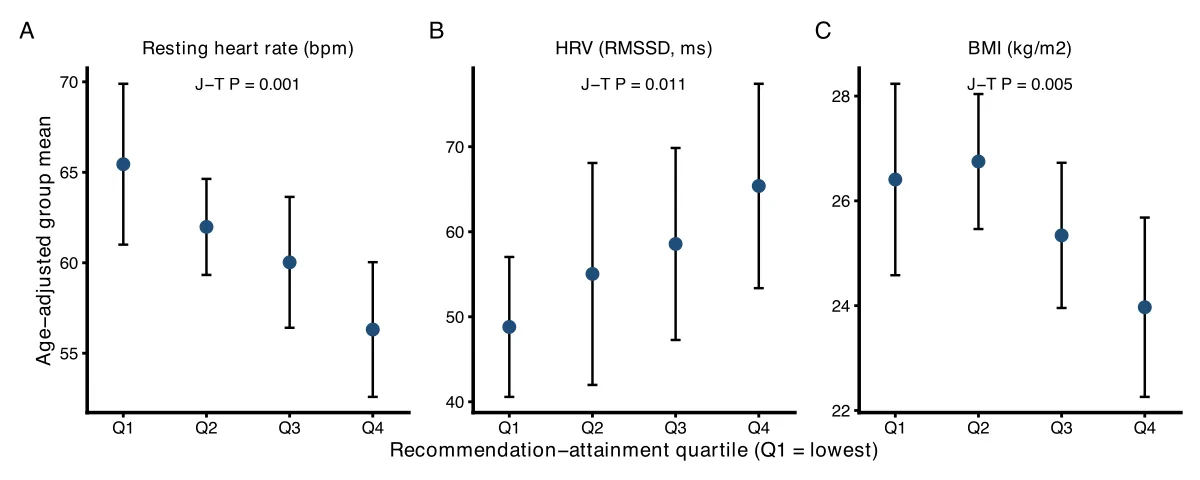
Age-adjusted cardiometabolic markers across recommendation-attainment quartiles. Panels show age-adjusted (A) resting heart rate, (B) heart rate variability (HRV) root mean square of successive differences, and (C) BMI across recommendation-attainment quartiles Q1 to Q4 (Q1=lowest attainment). Points are age-adjusted group means and error bars are 95% CIs; the Jonckheere-Terpstra (J-T) 2-sided trend *P* value is shown for each panel.

Prerevision recalculation reassigned 12 participants (15%) to an adjacent quartile (weighted κ=0.88) with similar effects (Table S2 in Multimedia Appendix 1). Strain-recommendation attainment accounted for the RHR and HRV gradients, whereas Sleep Performance alone was associated with neither; both contributed to the BMI gradient (Table S3 in Multimedia Appendix 1).

PMPY spending was US $62,042 in Q1 compared with US $6739, US $3091, and US $5656 in Q2 to Q4 (mean US $5162), approximately 12-fold higher than the other quarters; Q1 accounted for 80% of the cohort’s US $1.55 million total health care spending (Figure 2). Costs were concentrated in 6 catastrophic Q1 claim months that together comprised 84.9% of Q1 spending. When the largest month (US $307,311) was excluded, Q1’s PMPY spending was US $46,676 (~9-fold higher), and when all 6 of these catastrophic claim months were excluded, Q1’s PMPY spending was US $9394 (~1.8-fold higher).

**Figure 2.**
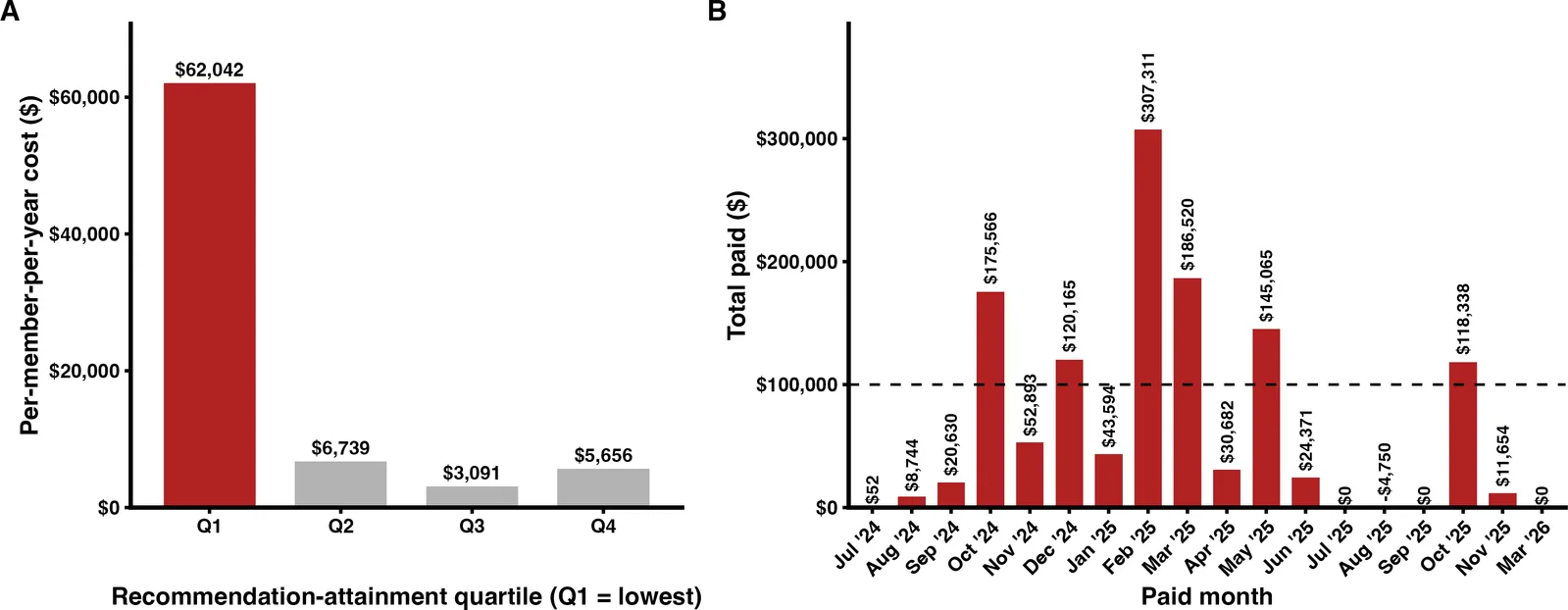
Concentration of health care spending by recommendation-attainment quartile. (A) Per-member-per-year cost (medical + pharmacy paid) by quartile; the lowest-attainment quartile (Q1, red) accounts for approximately 80% of total plan spend. (B) Q1 monthly paid totals ordered by calendar month, showing the distribution of high-cost months across the claims-payment period (dashed line = $100,000). Cost data were aggregated to the quartile level and are reported descriptively.

## Discussion

In this single-employer feasibility study, recommendation attainment was associated with more favorable cardiometabolic profiles, which supported construct validity of the metric, and linking wearable data with insurance claims was feasible at the group level, revealing a heavy concentration of spending in the lowest-attainment quartile.

This study has several limitations. The sample was small, drawn from a single employer, and predominantly male, limiting generalizability. Claims were at the quartile level only, and the cost difference was concentrated in 6 catastrophic claim months, which may reflect a small number of high-cost claimants and without which the Q1 excess fell from 12-fold higher to 1.8-fold higher. Because claim types were unknown, costs may reflect cardiometabolic disease, injury, acute illness, or unrelated expenses, and the cross-sectional design cannot exclude reverse causation, whereby acute or nonpreventable illness may have both reduced attainment and raised costs. Residual confounding by unmeasured baseline health status, medication use, occupational demands, and prior health care use could not be assessed. Finally, the Strain recommendation is calibrated to each member’s recent RHR, HRV, and sleep, so RHR and HRV are not fully independent criterion measures. Because this calibration is within person (recommendations are lower, and attainment easier, after above-baseline RHR nights), it should bias between-person associations toward the null, and the BMI gradient is algorithm independent. These findings are hypothesis generating and should not inform employment or insurance decisions. Nevertheless, this study demonstrates the feasibility of linking wearable-derived attainment metrics with physiology and claims and provides effect sizes to inform larger, longitudinal, multiemployer studies.

## Supplementary material

10.2196/109259 Multimedia Appendix 1 Supplementary Tables S1–S3: participant characteristics by attainment quartile, prerevision sleep performance sensitivity analysis, and component-specific associations with cardiometabolic outcomes.

## Abbreviations

HRV: heart rate variability
IRB: institutional review board
J-T: Jonckheere-Terpstra
PMPY: per-member-per-year (cost)
RHR: resting heart rate
