# Supplementary material for: Linking Attainment of Wearable-Derived Sleep and Physical Activity Recommendations with Cardiometabolic Biomarkers and Employer Health Care Claims: A Feasibility Study
Source: JMIR Form Res. 2026 Oct 22;10:e109259. doi: 10.2196/109259 (PMC13717263; doi:10.2196/109259)
Supplement: Supplementary file 1 [file formative-v10-e109259-s001.docx]

**Table S1**. Participant characteristics and cardiometabolic measures by physical activity and sleep recommendation attainment quartile (Q1 = lowest, Q4 = highest attainment).

| **Measure** | **Q1** | **Q2** | **Q3** | **Q4** | **Overall** |
| --- | --- | --- | --- | --- | --- |
| n | 20 | 20 | 20 | 20 | 80 |
| Age, y | 35.9 ± 9.2 | 29.8 ± 5.8 | 36.1 ± 10.5 | 35.0 ± 7.5 | 34.2 ± 8.7 |
| Female, % | 20 | 25 | 25 | 20 | 22.5 |
| BMI, kg/m² | 26.6 ± 4.4 | 26.1 ± 3.2 | 25.6 ± 3.5 | 24.1 ± 4.2 | 25.6 ± 3.9 |
| Wear, days | 250.9 ± 121.3 | 311.7 ± 82.1 | 292.5 ± 99.8 | 324.4 ± 55.4 | 294.9 ± 95.3 |
| Strain-recommendation attainment, % | 15.9 ± 12.4 | 28.0 ± 15.8 | 27.9 ± 14.7 | 46.4 ± 16.0 | 29.6 ± 18.2 |
| Sleep Performance, % | 73.6 ± 10.0 | 81.4 ± 8.5 | 84.1 ± 5.5 | 84.0 ± 8.0 | 80.8 ± 9.1 |
| RHR, bpm | 65.9 ± 10.5 | 60.9 ± 6.2 | 60.5 ± 8.3 | 56.5 ± 9.1 | 60.9 ± 9.1 |
| HRV, ms — median (IQR) | 46.4 (27.0–54.5) | 50.7 (40.0–80.5) | 56.1 (35.0–67.5) | 55.2 (47.3–83.1) | 50.3 (36.8–68.6) |

Values are mean ± SD unless otherwise noted. Strain-recommendation attainment and Sleep Performance were each adjusted for age and standardized before construction of the composite attainment score and quartile assignment; resting heart rate (RHR), heart rate variability (HRV), and body mass index (BMI) are outcome measures that did not enter the composite. HRV reported as median (IQR) and log transformed for inferential analysis because its distribution is right-skewed.

**Table S2**. Sensitivity analysis restricted to prerevision Sleep Performance data (mid-April 2025 revision): agreement between original and prerevision quartile assignments (A) and age-adjusted associations with cardiometabolic outcomes (B).

**A. Cross-tabulation of quartile assignment (n participants)**

| **Original quartile (full plan year)** | **Prerevision Q1** | **Prerevision Q2** | **Prerevision Q3** | **Prerevision Q4** | **Total** |
| --- | --- | --- | --- | --- | --- |
| Q1 (lowest attainment) | 18 | 2 | 0 | 0 | 20 |
| Q2 | 2 | 16 | 2 | 0 | 20 |
| Q3 | 0 | 2 | 16 | 2 | 20 |
| Q4 (highest attainment) | 0 | 0 | 2 | 18 | 20 |

**B. Age-adjusted associations with cardiometabolic outcomes**

| **Outcome** | **Full plan year: effect per SD (95% CI)** | **J-T P** | **Partial ρ (P)** | **Prerevision: effect per SD (95% CI)** | **J-T P** | **Partial ρ (P)** |
| --- | --- | --- | --- | --- | --- | --- |
| RHR, bpm | −3.6 (−5.4 to −1.8) | .001 | −0.34 (.002) | −3.2 (−5.1 to −1.4) | .002 | −0.33 (.003) |
| HRV, % (log scale) | +9.1 (−0.5 to +19.5) | .011 | 0.25 (.02) | +6.5 (−2.9 to +16.7) | .029 | 0.27 (.016) |
| BMI, kg/m² | −1.0 (−1.8 to −0.2) | .005 | −0.36 (.001) | −1.0 (−1.8 to −0.2) | .013 | −0.35 (.002) |

The prerevision analysis recalculated both composite components (Strain-recommendation attainment and Sleep Performance), their age adjustment and standardization, quartile assignment, and outcome means using only days before the mid-April 2025 revision (84.6% of person-days). Exact quartile agreement was 68/80 (85%); no participant moved by more than one quartile; linearly weighted κ = 0.88; Spearman ρ between original and prerevision composite scores = 0.98; 18 of 20 original Q1 members remained in Q1. Five participants had fewer than 45 prerevision days (range 7–31) and were retained. Effects are age-adjusted linear regression coefficients per SD of the composite score; HRV was modeled on the log scale and is expressed as percent change per SD. J-T, two-sided Jonckheere-Terpstra trend test across quartiles; partial ρ, age-adjusted partial Spearman correlation; RHR, resting heart rate; HRV, heart rate variability (RMSSD); BMI, body mass index; CI, confidence interval.

**Table S3**. Component-specific and mutually adjusted associations of Strain-recommendation attainment and Sleep Performance with cardiometabolic outcomes, compared with the composite recommendation-attainment score.

| **Exposure** | **RHR, bpm per SD (95% CI)** | **P** | **HRV, % per SD (95% CI)** | **P** | **BMI, kg/m² per SD (95% CI)** | **P** |
| --- | --- | --- | --- | --- | --- | --- |
| Composite score (primary analysis) | −3.6 (−5.4 to −1.8) | .001 | +9.1 (−0.5 to +19.5) | .011 | −1.0 (−1.8 to −0.2) | .005 |
| Strain-recommendation attainment only | −4.2 (−5.9 to −2.4) | <.001 | +18.5 (+8.8 to +29.0) | <.001 | −0.7 (−1.5 to +0.1) | .076 |
| Sleep Performance only | 0.0 (−2.0 to +2.0) | .30 | −6.7 (−14.9 to +2.3) | .043 | −0.5 (−1.3 to +0.4) | .17 |
| Mutually adjusted: Strain-recommendation attainment | −4.7 (−6.5 to −2.8) | <.001 | +17.9 (+7.7 to +29.1) | <.001 | −1.0 (−1.8 to −0.1) | .026 |
| Mutually adjusted: Sleep Performance | −1.5 (−3.4 to +0.3) | .10 | −1.4 (−10.0 to +7.9) | .75 | −0.8 (−1.7 to +0.1) | .064 |

Each exposure was age-adjusted and standardized (z-scored) in the same manner as the composite. Effects are age-adjusted linear regression coefficients per SD of the exposure; HRV was modeled on the log scale and is expressed as percent change per SD. For the composite and single-component rows, P is the two-sided Jonckheere-Terpstra trend test across quartiles of that exposure; for the mutually adjusted rows, both components were entered in the same age-adjusted regression and P is the regression P value. Age-adjusted partial Spearman correlations were −0.49 (RHR), 0.42 (HRV), and −0.22 (BMI) for Strain-recommendation attainment and 0.10, −0.17, and −0.17 for Sleep Performance. The two components were inversely correlated (age-adjusted r = −0.33). Claims data were aggregated by composite quartile and could not be analyzed by component. RHR, resting heart rate; HRV, heart rate variability (RMSSD); BMI, body mass index; CI, confidence interval.
